# Association of the TyG Index, Cardiometabolic Index, and Epicardial Adipose Tissue With Coronary Artery Disease

**DOI:** 10.1002/clc.70375

**Published:** 2026-06-11

**Authors:** Jia Min, Zhai Shumei, Zuo Yuqiang, Yin Yuling, Yang Xu, Xu Lei, Gao Zhihong

**Affiliations:** ^1^ Department of Cardiology The Second Hospital of Hebei Medical University Shijiazhuang Hebei People's Republic of China; ^2^ Department of Ultrasound North Campus, the Second Hospital of Hebei Medical University Shijiazhuang Hebei People's Republic of China; ^3^ Department of Physical Examination Center The Second Hospital of Hebei Medical University Shijiazhuang Hebei People's Republic of China; ^4^ Department of Cardiology Shijiazhuang People's Hospital Shijiazhuang Hebei People's Republic of China

**Keywords:** cardiometabolic index, coronary artery disease, epicardial adipose tissue, mediation analysis, triglyceride‐glucose index

## Abstract

**Objectives:**

Metabolic dysregulation and adiposity‐related alterations contribute to coronary atherosclerosis, yet the interrelations among cardiometabolic markers and epicardial adipose tissue index (EATi) remain unclear. This study aimed to investigate the associations of the triglyceride–glucose (TyG) index, cardiometabolic index (CMI), and EATi with coronary artery disease (CAD), and to explore potential metabolic–adiposity pathways.

**Methods:**

A total of 120 patients with CAD and 60 age‐ and sex‐matched controls were included. Clinical and biochemical data were collected. The TyG index and CMI were calculated, and EAT volume (EATV) was quantified by coronary computed tomography angiography (CCTA) and indexed to body surface area (EATi). Associations among variables were assessed using correlation analysis and multivariable logistic regression. Mediation and receiver operating characteristic (ROC) analyses were performed to assess potential pathways and diagnostic performance.

**Results:**

Fasting glucose, systolic blood pressure, triglycerides, TyG, CMI, and EATi were significantly higher in the CAD group (all *p* < 0.05). TyG (OR = 2.688, *p* = 0.002), CMI (OR = 3.339, *p* = 0.005), and EATi (OR = 2.284, *p* = 0.002) were independently associated with CAD. Mediation analysis demonstrated a significant indirect effect via CMI, whereas no mediation effect was observed for EATi. Combined assessment of TyG, CMI, and EATi demonstrated improved diagnostic performance for CAD (AUC = 0.847).

**Conclusion:**

The combined evaluation of TyG, CMI, and EATi improves the identification of CAD. CMI mediates the association between TyG and CAD, while EATi independently contributes to CAD risk, suggesting distinct metabolic and adiposity‐related mechanisms.

## Graphical abstract

This study demonstrates that the TyG index, CMI, and EATi are independently associated with CAD. CMI partially mediates the relationship between TyG and CAD, whereas EATi contributes through an independent pathway. These findings suggest that integrating metabolic and adiposity‐related markers may improve cardiovascular risk assessment.

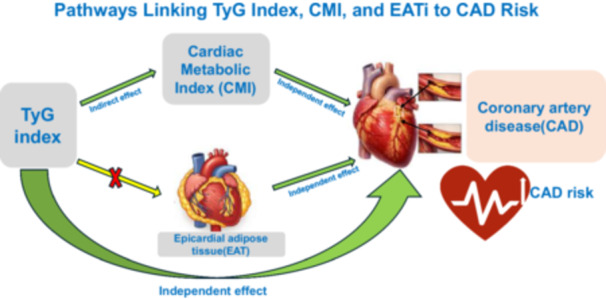

## 1 Introduction

Coronary artery disease (CAD) remains the leading cause of morbidity and mortality worldwide, continuing to impose substantial clinical and economic burdens despite ongoing advances in imaging, pharmacotherapy, and revascularization strategies [1]. The persistent risk of adverse cardiovascular events highlights the need for improved understanding of early cardiometabolic alterations that accompany coronary atherosclerosis, particularly among individuals without overt symptoms.

Epicardial adipose tissue (EAT), a metabolically active visceral fat depot surrounding the coronary arteries, has attracted increasing attention for its potential role in atherosclerotic disease. As an endocrine and paracrine organ, EAT secretes numerous cytokines, chemokines, and adipokines capable of influencing vascular inflammation and plaque vulnerability. Accumulating evidence demonstrates that increased EAT volume (EATV) or thickness is associated with the presence and severity of CAD, subclinical atherosclerosis, and high‐risk plaque features on coronary computed tomography angiography (CCTA) [2, 3, 4]. Given the close association of EATV with body size parameters such as body surface area (BSA) and BMI, the EAT index (EATi) provides a more standardized measure of epicardial adiposity.

In parallel, metabolic indices such as the triglyceride–glucose (TyG) index and cardiometabolic index (CMI) have emerged as simple, noninvasive surrogates for insulin resistance, visceral adiposity, and atherogenic dyslipidemia. The TyG index has been associated with coronary artery calcification, atherosclerotic burden, and cardiovascular events in diverse populations. CMI integrates the triglyceride‐to‐high‐density lipoprotein cholesterol (TG/HDL‐C) ratio with anthropometric parameters, such as waist‐to‐height ratio (WHtR), and has been validated as a sensitive marker of visceral adiposity and cardiovascular risk [5, 6, 7].

Although the associations of EATi, TyG, and CMI with CAD have been reported individually, the extent to which these markers may complement one another in reflecting coronary risk remains insufficiently explored. Recent studies suggest that integrating systemic metabolic markers with imaging‐derived indicators of pericoronary adiposity may offer complementary information about coronary atherosclerosis, particularly in individuals without overt clinical manifestations [8]. Furthermore, cardiometabolic derangement and epicardial fat accumulation may independently contribute to atherosclerosis progression through distinct yet complementary metabolic and inflammatory pathways.

Accordingly, this study aimed to investigate the associations of EATi, TyG index, and CMI with CAD and to explore potential metabolic–adiposity pathways through which these markers may independently relate to disease presence. By examining their combined performance and interrelationships, this study seeks to provide a multidimensional perspective on cardiometabolic alterations relevant to coronary atherosclerosis.

## 2 Materials and Methods

### 2.1 Data This Retrospective Case‐Control Study Included Patients Source and Study Population

This retrospective case–control study enrolled patients hospitalized at the Second Hospital of Hebei Medical University between June and December 2023 who were diagnosed with CAD based on the 2019 European Society of Cardiology Guidelines for the diagnosis and management of chronic coronary syndromes [9]. A total of 120 consecutive patients with CAD were enrolled in the case group. The control group consisted of 60 healthy individuals who underwent routine physical examinations at the hospital's health examination center during the same period, matched with the case group for age, gender, height, and weight. The mean age of the CAD group was 59.77 ± 7.25 years, with 74 males (61.7%), while the control group had a mean age of 59.40 ± 5.03 years, with 33 males (55.0%). Inclusion criteria were as follows ① age ≥ 18 years; ② CAD confirmed on CCTA (≥ 50% luminal stenosis); ③ matched controls undergoing routine health examinations with negative CCTA findings; ④ availability of complete clinical, biochemical, and imaging data. Exclusion criteria as follows: ① Presence of structural or congenital heart disease; ② History of heart failure or severe valvular disease; ③ Acute or chronic infections, malignancies, or autoimmune diseases; ④ Severe hepatic or renal dysfunction, or thyroid disease; ⑤ Poor‐quality CT images or incomplete EAT measurements; ⑥ Missing clinical records or laboratory biochemical parameters.

### 2.2 Data Collection

Baseline demographic and clinical information for all participants, including gender, age, height, weight, smoking history, alcohol consumption, and past medical history (cardiovascular disease, hypertension [HTN], diabetes mellitus [DM], and hyperlipidemia), as well as systolic blood pressure (SBP) and diastolic blood pressure (DBP), were collected from the electronic medical record system and the Health Management Center of the Second Hospital of Hebei Medical University.

Fasting venous blood samples (5 mL) were collected from each participant in the morning after at least 8 h of overnight fasting. Biochemical parameters, including total cholesterol (TC), triglycerides (TG), high‐density lipoprotein cholesterol (HDL‐C), low‐density lipoprotein cholesterol (LDL‐C), and fasting blood glucose (FBG), were measured using an automated biochemical analyzer in the hospital's central clinical laboratory following standard operating procedures.

### 2.3 Definitions

Body mass index (BMI) was calculated as: BMI = weight (kg) ÷ height^2^ (m^2^). The triglyceride‐glucose (TyG) index was calculated as ln (TG [mg/dL] × FBG [mg/dL]/2). Waist circumference (WC) was obtained from medical records. According to standard clinical practice, WC is measured in centimeters (cm) at the midpoint between the lower margin of the last palpable rib and the top of the iliac crest. WHtR was calculated as: WHtR = WC (cm) ÷ height (cm). CMI was calculated as: CMI = (TG [mmol/L] ÷ HDL‐C [mmol/L]) × WHtR. HTN was defined as SBP ≥ 140 mmHg, DBP > 90 mmHg, or ongoing treatment with antihypertensive medications. DM was defined as FBG level ≥ 126 mg/dL or treatment with hypoglycemic medications. Hyperlipidemia (HLD) was defined as TC ≥ 220 mg/dL, TG ≥ 150 mg/dL, LDL‐C ≥ 160 mg/dL, HDL‐C ≤ 40 mg/dL, or ongoing treatment with lipid‐lowering medications.

### 2.4 CCTA Examination

All CCTA examinations were performed using a dual‐source CT scanner (Somatom Definition Flash; Siemens Healthcare, Germany) with a gantry rotation time of 330 ms and a tube voltage of 120 kV. Patients with a heart rate > 60 beats/min received 25–75 mg of oral metoprolol (AstraZeneca, Shanghai, China) to maintain a target heart rate. All patients were given sublingual nitroglycerin immediately before scanning. Scans were performed in the supine position, covering the region from the tracheal carina down to the cardiac apex. After a preliminary coronary calcium scan, contrast volume was determined based on patient weight. Iopamidol, a nonionic contrast agent, was administered at 5.0–5.5 mL/s, followed by 40 mL of saline at the same rate. CCTA was acquired using a prospective or retrospective ECG‐gated protocol with a trigger threshold of 100 HU.

### 2.5 EATV Measurement

EATV was quantitatively measured from CCTA images on a dedicated workstation (Syngo.via VB20V, Siemens Healthcare, Germany). The measurement protocol followed our previously published methods [10, 11]. Briefly, EAT was defined as adipose tissue located between the myocardium and the visceral pericardium, with a CT attenuation threshold range from −190 to −30 Hounsfield units (HU), a range commonly used for fat segmentation that enables reliable discrimination from adjacent myocardium and contrast‐enhanced blood.

The measurement boundaries were set from the level of the pulmonary artery bifurcation to the apex of the left ventricle. The pericardial contour was manually delineated on axial images at 3–5 slice intervals. Given the anatomical continuity of the pericardium, this interval has been shown to provide reliable volumetric estimation while improving segmentation efficiency [12, 13]. The ROIs were then interpolated using a spline‐based segmentation algorithm based on a densitometric threshold. Adipose voxels within the defined attenuation range were then automatically identified and quantified to calculate the total EATV. EATi was calculated by dividing the measured EATV for each patient by the patient's BSA. BSA was calculated using a modified Stevenson formula: BSA (m^2^) = (84.4673 × height (cm)^0.6997 × weight (kg)^0.4176)/10,000, where height is expressed in centimeters and weight in kilograms.

All EATV measurements were independently performed by two experienced radiologists blinded to clinical and laboratory information; disagreements were adjudicated by a senior radiologist. Interobserver and intra‐observer reproducibility were assessed using intraclass correlation coefficients (ICCs) and Bland–Altman analysis.

### 2.6 Statistical Analysis

All statistical analyses were performed using SPSS version 22.0 (IBM Corp., Armonk, NY, USA). The distribution of continuous variables was assessed using the Kolmogorov–Smirnov test. Normally distributed variables (DBP, TC, TG, TyG index, and WHtR) were expressed as mean ± SD and compared using the independent samples *t*‐test. Non‐normally distributed variables (including EATi, age, height, weight, BMI, WC, SBP, FBG, CMI, HDL‐C, and LDL‐C) were presented as median and interquartile range (M [Q1, Q3]) and analyzed using the Mann–Whitney *U* test. Categorical variables were described as frequencies and percentages (*n*, %) and compared using the chi‐square (*χ* ^2^) test.

Pearson correlation analysis was performed to assess the relationships among the TyG index, EATi, and CMI. The coefficient of determination (*R* ^2^) was also calculated to reflect the proportion of variance explained. Multicollinearity was evaluated by calculating the tolerance and variance inflation factor (VIF), with VIF < 10 and tolerance > 0.1 indicating acceptable collinearity. To account for differences in measurement scales among the TyG index, CMI, and EATi, these variables were standardized using Z‐transformation prior to regression analyses. A multivariate logistic regression analysis with forward stepwise selection (Forward LR) was applied to identify independent predictors of CAD.

To explore mediating mechanisms linking the TyG index to CAD, parallel multiple mediation analysis was conducted using the PROCESS macro (Model 4). CMI and EATi were entered as parallel mediators. Indirect effects with 95% confidence intervals (CIs) were estimated via bias‐corrected bootstrapping with 5000 iterations. Receiver operating characteristic (ROC) curve analyses were performed to compare the predictive performance of TyG, CMI, EATi, and their combined model for CAD. All statistical tests were two‐tailed, with a *p*‐value < 0.05 considered statistically significant.

## 3 Results

**Figure 1:**
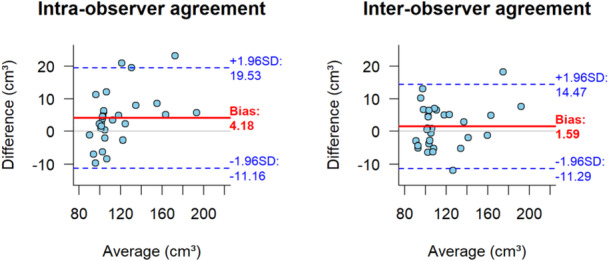
Bland‐Altman plots for EATV reproducibility.

The intra‐ and inter‐observer ICCs for EATV were 0.941 (95% CI: 0.848–0.974) and 0.968 (95% CI: 0.934–0.984), respectively. Bland‐Altman analysis demonstrated mean biases (95% LoA) of 4.18 (−11.16 to 19.53) cm^3^ and 1.59 (−11.29 to 14.47) cm^3^ for intra‐ and inter‐observer agreement, respectively (Figure 1).

A total of 180 subjects were enrolled in this study, including 120 patients with CAD and 60 healthy controls. Baseline demographic, anthropometric, and most clinical characteristics, including gender, age, BMI, and various cardiovascular risk factors, showed no significant differences between the CAD and control groups (*p* > 0.05 for all, Table 1), indicating good comparability between the two study populations. Compared with the control group, the CAD group showed significantly higher SBP (*p* = 0.037), FBG (*p* = 0.009), and TG levels (*p* < 0.001), while HDL‐C levels were significantly lower (*p* = 0.002). No significant differences were observed in TC or LDL‐C between the two groups (all *p* > 0.05).

**Table 1:** Comparative analysis of clinical, laboratory, and quantitative CT data between two groups of patients (*χ* ± SD or *M* [Q1, Q3]).

	All patients (*n* = 180)	CAD (*n* = 120)	Control (*n* = 60)	*t*/*Z*/*χ* ^2^	*p* value
Male gender (*n*, %)	107 (59.4)	74 (61.7)	33 (55.0)	0.737	0.390^a^
Age (year)	60.00 (56.00, 64.00)	60.50 (55.25, 65.00)	59.50 (56.00, 64.00)	0.635	0.525^b^
Height	167 (162, 171)	167 (160, 171.75)	168 (163, 171)	0.417	0.677^b^
Weight	70 (64, 78)	70 (64, 76)	71 (62.88, 80)	0.020	0.984^b^
BMI (kg/m^2^)	25.41 (23.63, 27.55)	25.39 (23.68, 27.55)	25.50 (23.00, 27.57)	0.563	0.573^b^
WC (cm)	89.73 (68.91, 99.06)	89.41 (69.82, 99.27)	90.12 (67.89, 98.06)	0.533	0.594^b^
WHtR	0.52 ± 0.09	0. 52 ± 0.09	0.51 ± 0.11	0.480	0.632^c^
Smoking (*n*, %)	130 (72.2)	88 (73.3)	42 (70.0)	0.222	0.638^a^
CAD history (*n*, %)	11 (5.7)	36 (30.0)	13 (21.7)	1.402	0.236^a^
Drinking history (*n*, %)	58 (32.2)	36 (30.0)	22 (36.7)	0.814	0.367^a^
HTN (*n*, %)	110 (61.1)	72 (60.0)	38 (63.3)	0.187	0.665^a^
DM (*n*, %)	72 (40.0)	56 (46.7)	16 (26.7)	3.110	0.078^a^
HLD (*n*, %)	76 (42.2)	52 (43.3)	24 (40.0)	0.182	0.670^a^
SBP (mmHg)	130 (120, 143)	130.00 (121.00, 145.00)	128.50 (119.25, 138.00)	2.082	0.037^b^ *
DBP (mmHg)	81.13 ± 10.84	82.16 ± 11.14	79.08 ± 9.98	1.804	0.073^c^
FBG (mmol/L)	6.14 (5.69, 6.83)	6.19 (5.74, 7.32)	5.94 (5.56, 6.42)	2.595	0.009^b^ *
TG (mmol/L)	1.54 ± 0.48	1.69 ± 0.45	1.24 ± 0.38	6.617	0.000^c^ **
HDL‐C (mmol/L)	1.13 (0.99, 1.33)	1.10 (0.91, 1.30)	1.20 (1.07, 1.40)	3.093	0.002^b^ *
LDL‐C (mmol/L)	2.75 (2.11, 3.50)	2.67 (1.02, 3.50)	3.03 (2.26, 3.49)	1.297	0.195^b^
TC (mmol/L)	4.47 ± 1.05	4.37 ± 1.15	4.64 ± 0.81	1.652	0.100^c^
TyG index	8.74 ± 0.40	8.88 ± 0.35	8.45 ± 0.32	7.784	0.000^c^ **
CMI	0.67 (0.46, 0.90)	0.79 (0.58, 0.95)	0.48 (0.36, 0.70)	5.714	0.000^b^ **
EATi (cm^3^/m^2^)	60.51 (55.77, 68.13)	63.84 (55.79, 71.25)	58.20 (55.76, 63.11)	2.819	0.005 ^b^ *

*Note:* a: *χ* ^2^ test, b: nonparametric rank sum test (Mann−Whitney *U* test), c: independent sample *t*‐test.

Abbreviations: BMI, body Mass Index; CMI, cardiometabolic risk index; DBP, diastolic blood pressure; DM, diabetes mellitus; EATi, epicardial adipose tissue volume index; FBG, fasting blood glucose; HDL‐C, high density lipoprotein cholesterol; HLD, hyperlipidemia; HTN, hypertension; LDL‐C, low density lipoprotein cholesterol; SBP, systolic blood pressure; TC, total cholesterol; TG, triglycerides; TyG index, triglyceride glucose index; WC, waist circumference; WHtR, waist‐to‐height ratio.

* *p* < 0.05;

** *p* < 0.001.

Regarding metabolic indices, the TyG index, CMI, and EATi were all significantly higher in the CAD group compared to the control group (all *p* ≤ 0.01, Table 1). Specifically, the TyG index was 8.88 ± 0.35 versus 8.45 ± 0.32; *t* = 7.784, *p* < 0.05, CMI was 0.79 (0.58, 0.95) vs 0.48 (0.36, 0.70) (*Z* = −5.714, *p* < 0.001), and EATi was 63.84 (55.79, 71.25) versus 58.20 (55.76, 63.11) cm^3^/m^2^ (*Z* = −2.819, *p* < 0.05).

### 3.1 Correlation and Collinearity Analysis Among Variables

To evaluate the intercorrelations among variables and assess the presence of multicollinearity within the multivariate logistic regression analysis, Pearson correlation analysis was first performed. The TyG index showed strong positive correlations with TG (*r* = 0.840, *p* < 0.01, adjusted *R* ^2^ = 0.704) and CMI (*r* = 0.607, *p* < 0.01, adjusted *R* ^2^ = 0.365). Furthermore, TG and CMI were highly correlated (*r* = 0.712, *p* < 0.01, adjusted *R* ^2^ = 0.504), while no statistically significant correlation was observed between EATi and other biochemical parameters (all *p* > 0.05) (Table 2). Subsequently, multicollinearity diagnostics were conducted within the multivariable logistic regression analysis. The VIF values for all independent variables were less than 10. Specifically, the VIF values were 1.593 for the TyG index, 1.583 for CMI, and 1.012 for EATi. In addition, all tolerance values exceeded 0.1, further confirming the stability and reliability of the regression analysis (Table 3).

**Table 2:** Pearson correlation coefficients among TyG index, CMI, EATi, and other metabolic parameters.

Variable	EATi	TG	TyG index	FBG	CMI
EATi	1	0.071	0.110	0.087	0.075
TG	0.071	1	0.840**	0.088	0.712**
TyG index	0.110	0.840**	1	0.580**	0.607**
FBG	0.087	0.088	0.580**	1	0.102
CMI	0.075	0.712**	0.607**	0.102	1

** *p* < 0.01 indicates statistical significance.

**Table 3:** Collinearity diagnostics for the multivariable logistic regression analysis.

Variable	Unstandardized coefficient (*B*)	SE	Standardized coefficient (Beta)	*t*‐value	*p* value	Tolerance	VIF
Constant	0.555	0.182		3.049	0.003		
EATi	0.007	0.002	0.182	2.851	0.005	0.988	1.012
TyG index	0.491	0.096	0.409	5.108	0.000	0.628	1.593
CMI	0.137	0.095	0.115	1.435	0.153	0.632	1.583

*Note:* Dependent variable = CHD group (1 = CHD, 0 = control). No multicollinearity detected (all VIF < 10 and Tolerance > 0.1).

### 3.2 Multivariate Logistic Regression Analysis for Predicting the CAD

Multivariate logistic regression analyses showed that the TyG index, CMI, and EATi were all significantly associated with CAD after mutual adjustment (Table 4). The TyG index demonstrated a robust association with CAD (OR = 2.688 95% CI: 1.421–5.084, *p* = 0.002). CMI also remained significantly associated with CAD (OR = 3.339, 95% CI: 1.431–7.789, *p* = 0.005). In addition, EATi, as an imaging‐derived marker of pericoronary fat burden, showed an independent association with CAD (OR = 2.284, 95% CI: 1.341–3.891, *p* = 0.002). The Nagelkerke *R* ^2^ for this multivariable model was 0.445. Sensitivity analyses revealed that the predictive value of EATi was highly robust; models using either absolute EATV or EATi (without BMI adjustment) yielded consistent effect estimates and identical directions of association (Supporting Information S1: Table 1). Furthermore, after further adjusting for BMI in the multivariable model, EATi remained a potent and independent predictor of CAD (OR = 2.341, 95% CI: 1.356–4.043, *p* = 0.002), whereas BMI itself showed no significant association with CAD risk (*p* = 0.599; Supporting Information S1: Table 1).

**Table 4:** Multivariate logistic regression analysis for independent predictors of CAD.

Variable	B	SE	OR (95% CI)	*p* value
TyG index	0.989	0.325	2.688 (1.421–5.084)	0.002
CMI	1.206	0.432	3.339 (1.431–7.789)	0.005
EATi	0.826	0.272	2.284 (1.341–3.891)	0.002

*Note:* All variables were standardized (*Z*‐scores) before inclusion in the model. *p* < 0.05 was considered statistically significant.

Abbreviations: B, regression coefficients; CI, confidence interval; OR, odds ratios; SE, standard errors.

### 3.3 Mediation Analysis

**Figure 2:**
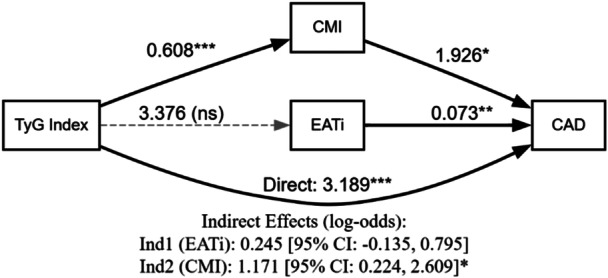
Mediation Analysis of CMI and EATi in the association between the TyG index and CAD (Model 4). Values on the paths are regression coefficients (B) expressed in a log‐odds metric. Solid lines represent significant pathways, while the dashed line indicates a non‐significant pathway (*p* > 0.05), *p < 0.05, **p < 0.01, ***p < 0.001.

A parallel multiple mediation analysis (Model 4) was conducted to examine the potential mediating roles of CMI and EATi in the association between the TyG index and CAD. The TyG index demonstrated a significant direct association with CAD (direct effect: 3.189, 95% CI: 1.707–4.671). Regarding indirect effects, a statistically significant mediation pathway was observed through CMI (*B* = 1.171, 95% CI: 0.224–2.609), whereas the pathway via EATi was not statistically significant (*B* = 0.245, 95% CI: –0.135–0.795). The total indirect effect was significant (*B* = 1.416, 95% CI: 0.331–3.052). Pairwise comparisons showed no significant difference between the CMI and EATi pathways (*B* = –0.926, 95% CI: –2.324–0.094) (Table 5, Figure 2).

**Table 5:** Parallel mediation analysis of TyG index on CAD via EATi and CMI.

Path number	Effect (log‐odds)	BootSE	95% CI
Direct effect	3.189	0.756	1.707−4.671
Ind 1	0.245	0.235	0.135−0.795
Ind 2	1.171	0.613	0.224−2.609
Total	1.416	0.688	0.331−3.052
(C1)	−0.926	0.623	2.324−0.094

*Note:* All effects are expressed in a log‐odds metric. Ind 1 = TyG → EATi → CAD; Ind 2 = TyG → CMI → CAD. The 95% CI was calculated using the percentile bootstrap method (5000 samples). An effect is considered statistically significant when the 95% CI does not contain zero.

Specifically, the TyG index was significantly associated with CMI (*B* = 0.608, *p* < 0.001), and CMI was significantly associated with CAD (*B* = 1.926, *p* = 0.033). In contrast, although EATi was significantly associated with CAD (*B* = 0.073, *p* = 0.001), its association with the TyG index was not statistically significant (*B* = 3.376, *p* = 0.141). (Supporting Information S1: Table 2, Figure 2).

### 3.4 Predictive Performance of TyG INDEX, CMI, EATI, and Their Combined Assessment for CAD

**Figure 3:**
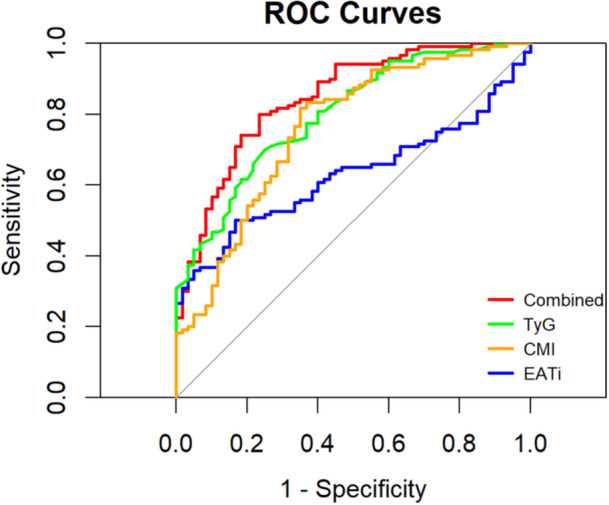
ROC curve analysis was performed to assess the discriminative power of individual predictors and their combined assessment for CAD. Combined represents TyG + EATi + CMI.

ROC curve analysis demonstrated that both the TyG index and CMI exhibited good discriminatory ability for CAD, with comparable performance (TyG: AUC = 0.801 [95% CI: 0.736–0.866]; CMI: AUC = 0.762 [95% CI: 0.685–0.838]; DeLong test, *Z* = 1.172, *p* = 0.2413). In contrast, EATi showed a significantly lower discriminative ability, AUC = 0.629 (95% CI: 0.550–0.708) (Figure 3).

When the three indicators were evaluated in combination, the discriminatory performance was further improved, yielding an AUC of 0.847 (95% CI: 0.788–0.906), with a sensitivity of 80.0% and a specificity of 76.7%. DeLong's tests indicated that the combined assessment was significantly superior to the TyG index alone (*Z* = 2.581, *p* = 0.0099), CMI alone (*Z* = 3.142, *p* = 0.0017), and EATi alone (*Z* = 4.945, *p* < 0.001). Additionally, both the TyG index and CMI demonstrated significantly better discriminatory performance than EATi (TyG vs. EATi: *Z* = 3.185, *p* = 0.0014; CMI vs. EATi: *Z* = 2.289, *p* = 0.0221).

## 4 Discussion

This study demonstrated that the TyG index, CMI, and EATi were all independently associated with CAD, although EATi was not significantly correlated with TyG or CMI. The combined evaluation of these markers improved the discrimination of CAD compared with each individual indicator (AUC = 0.847). The multivariable model incorporating these variables showed a Nagelkerke *R* ^2^ of 0.445, suggesting that these markers collectively account for a moderate proportion of the variance in CAD risk.

Mediation analysis further suggested that CMI may partially mediate the association between the TyG index and CAD, whereas EATi is independently associated with CAD. The absence of a significant indirect effect through the EATi pathway indicates that metabolic and adiposity‐related measures contribute to CAD through partially independent mechanisms. These findings suggest that systemic cardiometabolic dysfunction and epicardial fat accumulation may operate through parallel, rather than strictly sequential, pathways in the development of coronary atherosclerosis.

Consistent with this framework, patients with CAD in the present study exhibited significantly higher TyG index and CMI values, reflecting the presence of metabolic dysregulation and visceral adiposity commonly observed in atherosclerotic disease. Both indices have been widely reported as indicators of insulin resistance, dyslipidemia, and adverse cardiometabolic profiles, which are closely linked to the development and progression of coronary atherosclerosis [5].

The TyG index, derived from the product of fasting plasma glucose and triglyceride levels, has been recognized as a reliable surrogate marker for IR and metabolic dysfunction. In this study, the TyG index demonstrated a significant and independent association with both the presence of CAD. Each 1‐standard‐deviation increase in TyG was associated with markedly higher odds of having CAD, supporting its relevance to cardiometabolic disturbances that accompany atherosclerotic processes. These findings are consistent with previous research showing that elevated TyG values correlate with coronary artery calcification, overall atherosclerotic burden, and adverse cardiovascular outcomes [14, 15, 16].

Recent observational and cohort studies further reinforce these associations. For example, Liu et al. reported that higher TyG levels were linked to increased cardiovascular events and all‐cause mortality, independent of traditional risk factors [17]. Other studies have shown that TyG is related not only to the presence of coronary plaques but also to plaque severity, including multivessel involvement and complex lesion characteristics [18].

Several biological mechanisms may underlie these observations. Insulin resistance is known to reduce nitric oxide bioavailability, impair endothelial function, and promote oxidative stress and low‐grade inflammation. It also contributes to dysregulated lipid metabolism, including elevated VLDL and reduced HDL concentrations, which collectively facilitate lipid infiltration, macrophage recruitment, and foam‐cell formation—early hallmarks of atherosclerotic plaque development. These interconnected pathways help explain the observed relationship between TyG and CAD in this study [19, 20, 21].

Taken together, the TyG index appears to reflect key metabolic disturbances relevant to coronary atherosclerosis and may provide clinically useful information in understanding cardiometabolic alterations among individuals at risk for CAD [22]. While our findings support its potential value, further prospective studies are necessary to clarify its role across diverse populations and to better define how TyG may contribute to a broader, multidimensional assessment of cardiometabolic health.

The CMI, which integrates lipid metabolism through the triglyceride‐to‐HDL cholesterol ratio and central adiposity via the waist‐to‐height, has gained increasing recognition as a comprehensive indicator of metabolic burden and cardiovascular risk [23]. As a composite measure, CMI reflects the combined effects of dyslipidemia and visceral adiposity—two key contributors to cardiometabolic dysfunction. Elevated CMI levels have been associated with HTN, type 2 diabetes, dyslipidemia, and non‐alcoholic fatty liver disease (NAFLD) in multiple populations, underscoring its relevance as a marker of systemic metabolic disturbance [24, 25, 26].

In the present study, CMI demonstrated an independent association with CAD. A one‐standard‐deviation increase in CMI was associated with a 3.34‐fold higher likelihood of CAD, supporting its ability to capture the synergistic influence of visceral adiposity and metabolic dysregulation on atherogenesis. This finding aligns with previous research showing that higher CMI levels correlate with CAD presence even after adjustment for traditional cardiovascular risk factors [27]. Furthermore, mediation analysis identified CMI as a key intermediary in the association between the TyG index and CAD, suggesting that metabolic abnormalities reflected by TyG may promote atherosclerosis partially through pathways linked to central adiposity. This highlights the potential value of CMI in characterizing metabolic–adiposity interactions relevant to coronary disease.

EAT has also emerged as a critical contributor to CAD pathogenesis because of its close anatomical proximity to the coronary arteries and its capacity to secrete inflammatory adipokines. Functionally, EAT is a metabolically active organ that secretes pro‐inflammatory cytokines and adipokines, which directly impact coronary vasculature and contribute to atherosclerosis [28, 29]. Previous studies have demonstrated consistent associations between increased EATV and both the presence and severity of CAD [30, 31]. Consistently, EATi was significantly higher among patients with CAD, supporting the concept that EAT is not merely a marker of adiposity but a metabolically active tissue involved in local vascular inflammation and plaque development.

In addition, EATi showed no significant correlation with the TyG index or CMI, suggesting that EATi may act as a relatively distinct pathogenic component rather than a mere downstream manifestation of systemic metabolic and adiposity disturbances. Consistently, EATi did not significantly mediate the TyG–CAD pathway. This lack of association might be explained by the complex, non‐linear relationship between insulin resistance and epicardial fat. EATi may promote atherosclerosis through mechanisms independent of systemic metabolism, such as localized paracrine signaling and direct anatomical interactions with coronary vessels. Recent evidence linking EAT to coronary microvascular dysfunction [32] provides a plausible perspective on the independent deleterious impact of EATi observed here. Finally, qualitative features of epicardial fat, such as inflammatory activity, may have greater implications for coronary plaque vulnerability than fat volume alone [33, 34]. a factor not assessed in this study but worthy of further investigation.

## 5 Limitations of the Study

This study has several limitations. First, its retrospective case–control design prevents causal inference, and the observed associations should be interpreted as exploratory. Although multivariable adjustments were performed to minimize confounding, strict 1:1 matching between cases and controls was not applied. Prospective studies with more rigorous matching strategies are needed to further validate these findings and clarify the temporal relationships among TyG, CMI, EATi, and CAD. Second, the study population consisted of hospitalized patients with CAD, which may limit the generalizability of the findings to broader or asymptomatic populations. Larger and more diverse cohorts would improve external validity. Third, EATi was assessed using CCTA, which quantifies fat volume but does not capture qualitative inflammatory or compositional features that may also influence CAD risk. Additional imaging modalities could provide a more comprehensive evaluation. Fourth, anthropometric data, particularly WC, were derived from routine clinical records and may be subject to variability in measurement and recording. Therefore, WC‐related indices should be interpreted with caution. Finally, other potentially relevant biomarkers—such as inflammatory cytokines or genetic factors—were not assessed. Future studies incorporating a wider range of metabolic, inflammatory, and imaging indicators may help refine the understanding of cardiometabolic pathways related to CAD.

## 6 Future Research Directions

Future studies should employ longitudinal designs to clarify the temporal relationships among TyG, CMI, EATi, and CAD progression, as the present cross‐sectional analysis cannot establish causality. More refined imaging approaches—such as MRI or PET—may help characterize qualitative features of epicardial fat and further validate its role in coronary atherosclerosis. In addition, interventional studies targeting metabolic dysfunction or reducing epicardial fat, including pharmacologic or lifestyle strategies, are warranted to determine whether modifying these markers can reduce CAD risk. These efforts may ultimately contribute to more precise and individualized cardiovascular prevention strategies.

## 7 Conclusions

This study demonstrates that the TyG index, CMI, and EATi are each independently associated with obstructive CAD, and that their combined assessment improves the identification of disease presence. The observed statistical pathways suggest that metabolic and adiposity‐related disturbances may contribute to coronary atherosclerosis through partially independent mechanisms, with CMI potentially acting as an intermediary link between systemic metabolic dysfunction and CAD, whereas EATi appears to be independently associated with disease rather than serving as a downstream mediator. Collectively, these findings suggest the potential value of integrated metabolic and adiposity markers in CAD assessment, pending validation in prospective, multicenter studies.

## Author Contributions

Gao Z.H. was responsible for conceptualization, supervision, project administration, and critical review of the manuscript. Yin Y.L. and Zuo Y.Q. performed the methodology, software, formal analysis, data curation, and visualization. Yang X. and Xu L. handled the investigation and resources. Jia M. and Zhai S.M. prepared the original draft. Jia M. and Zhai S.M. contributed equally to this work. All authors contributed to data interpretation, critically revised the manuscript for important intellectual content, and approved the final version for submission.

## Funding

The authors have nothing to report.

## Ethics Statement

This retrospective study was approved by the Research Ethics Committee of the Second Hospital of Hebei Medical University (Approval No. 2025‐R206). The requirement for informed consent was waived due to the retrospective nature of the study, in accordance with national legislation and institutional requirements.

## Conflicts of Interest

The authors declare no conflicts of interest.

## Supporting information


**Supporting File:** clc70375‐sup‐0001‐Supplementary_Materials.docx.

## Data Availability

The data supporting the findings of this study are available from the corresponding author upon reasonable request.
