# Supplementary material for: Association of the TyG Index, Cardiometabolic Index, and Epicardial Adipose Tissue With Coronary Artery Disease
Source: Clin Cardiol. 2026 Jun 11;49(6):e70375. doi: 10.1002/clc.70375 (PMC13255140; doi:10.1002/clc.70375)
Supplement: Supplementary file 1 — Supporting File: clc70375‐sup‐0001‐Supplementary_Materials.docx. [file CLC-49-e70375-s001.docx]

**Supplementary Materials**

**Table S1. Multivariable logistic regression analyses evaluating the association between EAT and CAD under different modeling strategies**

|  | Variable | OR (95% CI) | P value |  |
| --- | --- | --- | --- | --- |
| Model 1 EATV | TyG index | 2.747 (1.442–5.234) | 0.002 | |
|  | CMI | 3.047 (1.310–7.086) | 0.010 | |
|  | EATV | 2.169 (1.231–3.824) | 0.007 | |
| Model 2 EATi | TyG index | 2.688 (1.421–5.084) | 0.002 | |
|  | CMI | 3.339 (1.431–7.789) | 0.005 | |
|  | EATi | 2.284 (1.341–3.891) | 0.002 | |

| Model 3 EATi + BMI* | TyG index | 2.663 (1.405–5.048) | 0.003 | |
| --- | --- | --- | --- | --- |
|  | CMI | 3.387 (1.449–7.918) | 0.005 | |
|  | EATi | 2.341 (1.356–4.043) | 0.002 | |
|  | BMI | 0.898 (0.601–1.342) | | 0.599 |

Abbreviations: CAD, coronary artery disease; TyG, triglyceride–glucose index; CMI, cardiometabolic index; EATV, epicardial adipose tissue volume; EATi, EATV indexed to body surface area; BMI, body mass index; CI, confidence interval; OR, odds ratio.

* Model 3 represents a sensitivity analysis with additional adjustment for BMI.

All variables were standardized using Z-score transformation prior to entry into the logistic regression model. P < 0.05 was considered statistically significant.

**Table S2. Path coefficients and model fit for the parallel mediation analysis.**

| Outcome Variable | Predictor | Coeff | SE | t/Z | P |
| --- | --- | --- | --- | --- | --- |
| EATi (R^2^ = 0.012) | Constant | 58.495 | 3.253 | 17.985 | < 0.001 |
|  | TyG (Path a_1_) | 3.376 | 2.286 | 1.477 | 0.141 |
| CMI (R^2^ = 0.368) | Constant | -0.089 | 0.085 | -1.051 | 0.295 |
|  | TyG (Path a_2_) | 0.608 | 0.060 | 10.182 | < 0.001 |
| CAD (group) (Nagelkerke  R^2^ = 0.445) | Constant | -9.128 | 1.852 | -4.928 | < 0.001 |
|  | TyG (Path c') | 3.189 | 0.756 | 4.218 | < 0.001 |
|  | EATi (Path b_1_) | 0.073 | 0.023 | 3.202 | 0.001 |
|  | CMI (Path b_2_) | 1.926 | 2.137 | 2.137 | 0.033 |

Note: a represents the path from the independent variable (TyG) to the mediator; b represents the path from the mediator to the outcome (CAD); c' represents the direct effect. t values are derived from ordinary least squares regression (mediator models), and Wald Z values are derived from logistic regression (outcome model). All coefficients are unstandardized, with the outcome model expressed in a log-odds metric.
